# A Survey for Examining the Effects of COVID-19 and Infection Control Measures in Older Persons With Mild Cognitive Impairment and Dementia and Their Caregivers

**DOI:** 10.3389/fpsyt.2020.599851

**Published:** 2020-11-16

**Authors:** Desirée E. Porcari, Katie Palmer, Gianfranco Spalletta, Valentina Ciullo, Nerisa Banaj

**Affiliations:** ^1^Laboratory of Neuropsychiatry, Department of Clinical and Behavioral Neurology, IRCCS Santa Lucia Foundation, Rome, Italy; ^2^Department of Neuroscience, University of Rome Tor Vergata, Rome, Italy; ^3^Department of Internal Medicine and Geriatrics, Università Cattolica del Sacro Cuore, Rome, Italy; ^4^Menninger Department of Psychiatry and Behavioral Sciences, Baylor College of Medicine, Houston, TX, United States

**Keywords:** COVID-19, SARS-CoV-2 coronavirus, neurocognitive disorders, mild cognitive impairment, MCI, dementia, caregivers, neuropsychiatric symptoms

## Abstract

**Background:** During the first wave of the COVID-19 pandemic, many non-urgent outpatient services in Italy were closed due to the Government-enforced lockdown period. So far, little is known about what effect the pandemic, quarantine measures, and reductions in medical services had on people with cognitive impairment and their caregivers.

**Objectives:** To develop two versions (i.e., patients and informants/caregivers) of a survey designed to assess the impact of the COVID-19 pandemic during the first Italian lockdown period (11 March −4 May 2020) on Memory Clinic outpatients with Mild Cognitive Impairment (MCI) or dementia, and their caregivers.

**Design:** Psychiatrists, neuropsychologists, and epidemiologists developed two versions: one for patients with Mild Cognitive Impairment and other cognitive disorders, the other for their relatives and/or caregivers. Each version of the survey includes five sections: (a) socio-demographic information and access to technology devices; (b) individual COVID-19 protection methods; (c) knowledge about COVID-19; (d) the effect of COVID-19 on daily life; and (e) the effect of COVID-19 on emotional state.

**Conclusion:** Until an effective vaccine is developed it is likely that future waves of COVID-19 will result in shielding of vulnerable older adults. We believe that this instrument will be useful as a tool to collect information and help clinicians to promptly respond to changes in patients' cognitive, psychiatric, and somatic health needs, and to help for future planning in possible subsequent quarantine periods.

## Introduction

Since the first confirmed case of SARS-CoV-2 infection was reported in Wuhan China in December 2019 (1), the worldwide pandemic has already caused thousands of deaths in several countries, including Italy, which was one of the first European countries to be seriously affected (2, 3).

On 11 March 2020, the Italian Government implemented a series of measures to contain the spread of the virus including unprecedented levels and scales of quarantine, physical distancing, and community lockdowns with people staying at home, leaving only for essential services or activities (e.g., travel to work for essential workers, grocery shopping, urgent medical care etc.). In addition, many non-urgent outpatient services were closed and appointments canceled or postponed in order to reduce the risk of infection in patients and healthcare personnel and to ease burden on the national healthcare service (4). Many countries have employed similar lockdown and social distancing measures (5). Although this focus on procedures to urgently slow SARS-CoV-2 infection rates and minimize the number of infected individuals is of utmost importance, it also has relevant implications for the short—and long -term health and well-being of patients with non-communicable diseases (6), including neurological and psychiatric conditions. It is possible that patients with pre-existing clinical conditions may experience a worsening of their symptoms or an increase in disease progression in relation to some of the infection containment measures (e.g., due to lack of exercise, social isolation, etc.).

Patients with Mild and Major Neurocognitive Disorders [e.g., Mild Cognitive Impairment (MCI), Alzheimer Disease (AD)] are particularly vulnerable in the ongoing pandemic due to the high prevalence of chronic diseases and disabilities. These individuals have experienced a change in access to both formal and informal care during the pandemic. Additionally, the objective evidence of cognitive impairment (i.e., memory, executive function), may cause difficulties in following safeguarding procedures (such as wearing masks, social distancing, and hygiene) or in understanding the public health information issued to them, thus generating anxiety and emotional distress for both themselves and their caregivers. In patients with Neurocognitive Disorders an association between neuropsychiatric symptoms and lack of cognitive stimulation (7–9) and social isolation (10–12) have been shown. During periods of confinement, informal caregivers and family members may also experience management, economic, and personal difficulties (13, 14).

Since the beginning of the pandemic, the scientific community has made great efforts to investigate and document the possible effects of government containment measures on the population but evidence is sparse. Most studies focused on describing the effects of the pandemic on the general young and adult population, showing worsening in mood, anxiety, and manifestations similar to Post-traumatic Stress Disorder (15–21). Conflicting data also emerged from studies exploring the effect of confinement on quality of life and mental health in patients' with Neurocognitive Disorders. While one research group (22) reported that only a small percentage of people with AD experienced worsening of cognitive and neuropsychiatric aspects, others observed significative worsening of neuropsychiatric symptoms (i.e., agitation, apathy, and aberrant motor activity) without a decrease in quality of life in either patients and caregivers (23). These conflicting results may be due to methodological differences and to the fact that, in some cases, the authors remotely administered interviews that were not specifically developed for the ongoing pandemic. Consequently, instruments that specifically allow an overall and comparable assessment of the impact of the pandemic on patients affected by Neurocognitive Disorders and their caregivers are urgently needed.

Here we describe two versions of a survey to assess the impact of the COVID-19 pandemic during the first Italian lockdown period (11 March−4 May 2020) on Memory Clinic outpatients and their caregivers. Due to the risk of infection to staff and patients, the survey was developed to be administered remotely (e.g., by phone call) to ensure that characteristics of patients could be accurately documented during the lockdown period, without the need for face-to-face contact. We show our study protocol and the structure of the survey, to allow other research groups to use it or adapted it according to cultural characteristics.

The primary aim of the study was to develop a survey evaluating: (i) patients' knowledge of COVID-19 and recommended hygiene procedures, (ii) barriers that these patients face during lockdowns in terms of infection control, such as lack of masks and disinfectant gel, and physical health, such as access to outdoor space for exercise, (iii) effects of lockdown procedures on access to medical care, prescription drugs, and informal care, (iv) mood and other behaviors of patients and caregivers during periods of lockdown, including depressive symptoms, anxiety, and problems sleeping, (v) effects of confinement on patients, and how this affects caregivers, and vice versa, and (vi) factors (lifestyle, living situation etc.) that correlate with mood in both caregivers and patients.

## Methods and Analyses

### Development of the Survey

The “Effect of the COVID-19 Lockdown on Persons with Neurocognitive Disorders and their Caregivers Survey” was developed to comprehensively assess the impact of the COVID-19 lockdown on outpatients with MCI, dementia, and their caregivers. The survey has two versions: (i) patients and (ii) informants/caregivers. The latter was formulated to collect information about the informant/caregivers themselves as well as the older person that they were taking care of. The survey was developed by a group of experts specialized in Neurocognitive Disorders, including Geriatric Psychiatrists, Neuropsychologists, and Epidemiologists. They established crucial research questions of interest during the pandemic (e.g., What is the level of knowledge about COVID-19, protection methods and infection control measures etc.? What was the impact of the lockdown on the medical appointments and medication availability? Did the pandemic have an effect on daily activities? Did patients and caregivers express symptoms of anxiety, stress, depressed mood and other symptoms during lockdown? etc.).

It consists of five sections: (a) general information, including socio-demographic information and access to technology devices; (b) individual protection methods; (c) knowledge about COVID-19; (d) the effect of COVID-19 on daily life and (e) the effect of COVID-19 on emotional state. It includes novel questions devised to assess the new pandemic, as well as questions adapted from existing scales (24, 25). The survey was designed using language that was familiar to lay persons (e.g., using terms such as “coronavirus” etc.).

The “General Information” section in the patient version of the survey provides socio-demographic information about older adults, with a focus on their accommodation type, with whom they lived during the quarantine and collects information on tobacco and alcohol consumption (from questions 1 to 8). We included specific questions related to the environment of the individuals, including whether they had access to outdoor space, and what type of technology they had access to for communicating with family and friends. These questions were designed to identify whether any environmental factors were related to emotional state.

“Individual protection methods” (questions 9–11), assesses type of individual protection methods (e.g., if they have a surgical mask, if was difficult to obtain one, how often they wash their hands, and for how long). These questions were designed to assess whether there are any practical limitations that may lead to problems following Government guidelines (e.g., lack of face mask availability or cognitive difficulties in understanding regulations).

The third section, “Knowledge about COVID-19” (questions 12–16) investigates how well the patients kept themselves informed about COVID-19 and Government regulations through media.

The section “The effect of COVID-19 on daily life” (questions 17–31) is the core of the survey. It ranges from questions about concerns of COVID-19 (fear of infection and type of symptoms experienced in case of illness), how individuals changes their daily routines due to confinement, physical activities, help in basic and instrumental activities of daily living, changes in drug intake (e.g., due to forgetfulness), medical visits missed, difficulties in purchasing medication, type and frequency of communication with relatives and friends.

The last section “The effect of COVID-19 on emotional state” consist of 23 questions in both patients and caregivers; the informant version includes questions about distress-burnout.

The caregivers' version investigates similar aspects, with a focus both on the caregiver and patient; in this case the caregiver expresses their opinion on how the patient has coped with confinement and quarantine measures.

The survey was edited both in Italian and English. Both versions were translated and back-translated by native speakers. The complete survey is provided in Appendices 1–4.

### Design of the Ongoing Study

We conducted an observational study using the two versions of the survey. To determine the sufficient sample size, a power analysis was conducted using G^*^Power with an alpha of 0.05, a power of 0.95, an effect size of 0.35 and a predictor number of 10. Based on the aforementioned assumptions, the desired total sample size was 80. Considering that comparable studies for dementia cohorts with a sample size > 100 showed efficacy in describing neuropsychiatric phenomena, we contacted 150 patients and 150 caregivers (26–28).

Patients and their caregivers were identified using a large, established research database from the outpatient Memory Clinic at Santa Lucia Foundation IRCSS, which has been used for previous studies (29–31). All patients referred to our clinic underwent extensive neurological, neuropsychiatric, and cognitive testing and diagnosis was made according to international diagnostic criteria (32). Patients who participated in previous research studies and who gave their permission to be contacted for future studies, were phoned by the research team (neuropsychologists) to ask if they would like to participate in the new survey. Those consenting to participate in the study were asked if they agree for their caregiver to be contacted. Caregiver has been defined as a family member, friend, or other, who undertakes unpaid care in and assistance in activities of daily living (child, spouse, etc.) (33). Data collected during the first Italian lockdown period will be used in future works.

## Ethics and Procedures

The Santa Lucia Foundation ethical review board approved the study protocol (code number CE/PROG.827). Prior to the administration of the survey, we fully informed participants about the study design, purposes and type of involvement required, specifying that they could withdraw from participation at any time. They had the opportunity to ask questions and they received a copy of the informed consent by post. In order to minimize unnecessary face-to-face contact and to adhere to Government restrictions, the survey was administered remotely (i.e., by phone call) by a trained psychologist or physician. The time of administration was ~25 min.

## Discussion

The aim of this paper was to describe a comprehensive survey that was developed to assess specific aspects of the COVID-19 pandemic and infection control measures in patients with Neurocognitive Disorders and their caregivers. We aimed at providing a comprehensive instrument that can assess multiple consequences of the pandemic. Before the pandemic, there were no scales that could accurately assess the novel characteristics that individuals now face in relation to lockdown measures, such as whether patients have access to outdoor space for exercise and whether there are any changes in the amount of informal and formal care received. Importantly, our survey aimed at assessing how these factors may affect the mood and other neuropsychiatric behaviors of both patients and caregivers. Furthermore, the survey includes a specific section on caregiver distress and provides information on their point of view of how the patient is coping. Another novel aspect is the investigation of practical aspects that may have great importance in these patients, such as the effect of the lockdown access to medical appointments and treatments. Government regulations, restrictive measures, and other aspects of the pandemic are continually changing, making it challenging to develop a survey that will be fully relevant in the long term. For example, as new outbreaks occur, it is likely that some restrictive measures differ from the first wave of the pandemic. However, we believe that the present survey will be a useful tool to collect information about possible changes in patients' status and help clinicians to promptly respond to changes in patient's health.

The COVID-19 pandemic makes it important to design specific instruments to assess consequences which have never been experienced before. In addition, to assessing changes in patients' cognitive and mental health symptoms, our survey also provides important assessment of the secondary consequences of infection control measures, such as reduced access to medical services or difficulties getting prescriptions, and practical limitations that were faced by many people during the pandemic, such as access to reliable information on COVID-19 and lack of available infection protective equipment, such as masks and gloves. Responses to these questions could be relevant in the near future, when additional peaks or further waves of COVID-19 are highly possible. Policy makers may need to consider such limitations when planning subsequent lockdowns. The survey can also be used in the event of another lockdown period, to assess changes in patient and caregivers' status in comparison to the first wave of COVID-19.

Some limitations of the survey should be discussed. The pandemic is a completely novel event, and Italy was one of the first countries to be badly affected. It was important to act promptly because the restrictive measures on research and clinical activities by government regulations made it impossible to create a focus group on site with patient and caregiver advocates. Thus, the survey was not developed in conjunction with either patients or caregivers. However, a consensus meeting with a group of psychiatrists, neuropsychologists, and epidemiologists with extensive expertise on the target group was conducted. Another aspect to be considered is the lack of validated COVID-19 pandemic scales during the lockdown. Further, the COVID-19 pandemic is a rapidly evolving situation and it was challenging to develop a survey in the early stages of the pandemic, capturing all aspects that might affect patients with neurocognitive disorders in the first and subsequent potential COVID-19 outbreaks. There has been an unprecedented increase of scientific publications on the topic of COVID-19 (34–37), and evidence is emerging daily. For example, several publications have now indicated that there might be a form of post-traumatic stress disorder directly related to the virus and the lockdown scenarios (17, 20), yet our survey did not directly assess this issue. Our survey was designed to get a picture of the situation faced by memory clinic outpatients during the first wave of the pandemic, when only telephone assessment was possible, which limited the possibility to diagnose precisely complex mental disorders. However, focusing mainly on dimensional phenomenology, as we did, may capture fundamental aspect of status psychopathology.

## Dissemination

In future stages, we will describe the results collected using the survey in our Memory Clinic patients, who are already involved in other research projects (8, 29, 30, 38). We aim to compare the results of our survey with other assessments both in Italy and other countries. The two specific versions of the survey will allow to assess how patient characteristics affected caregiver status during the quarantine period and will hopefully highlight issues that need to be addressed in future outbreaks. Further, the rich pre-pandemic dataset from our memory clinic will allow to assess changes in mood and other neuropsychiatric characteristics during the quarantine period comparing to pre-pandemic status. Other secondary objectives that will be clarified include: how caregiver burden during the lockdown correlates to specific factors (e.g., increased patient stress) and how stress levels differed between caregivers living with and those separately from patients.

Until an effective vaccine is developed it is likely that future waves of COVID-19 will mostly affect vulnerable older adults, and we are confident that our survey will help to provide information to better protect them and their caregivers.

## Author Contributions

GS, NB, and KP conceived the study protocol and the structure and content of the paper. DP, NB, and KP wrote the paper. NB, VC, and GS provided revisions of the paper. All authors contributed to the article and approved the submitted version.

## Conflict of Interest

The authors declare that the research was conducted in the absence of any commercial or financial relationships that could be construed as a potential conflict of interest.
