# Supplementary material for: A Survey for Examining the Effects of COVID-19 and Infection Control Measures in Older Persons With Mild Cognitive Impairment and Dementia and Their Caregivers
Source: Front Psychiatry. 2020 Nov 16;11:599851. doi: 10.3389/fpsyt.2020.599851 (PMC7701287; doi:10.3389/fpsyt.2020.599851)
Supplement: Supplementary file 1 [file Data_Sheet_1.pdf]

Appendix 1. The Effect of the COVID-19 Lockdown on Persons with Neurocognitive Disorders – Patient version

Identification code: ___________ **Date: ___ / ___ / ____**

THE EFFECT OF THE COVID-19 LOCKDOWN ON PERSONS WITH NEUROCOGNITIVE DISORDERS PATIENT VERSION

The purpose of this survey is to obtain information about your experience of the lockdown that has occurred because of the coronavirus pandemic. The questions are not designed to check up on you to see if you are following any rules - none of the information you provide will be given to anyone else. The questionnaire is completely anonymous and subject to the European and National privacy regulations (UE 2016/679). Your data will be used exclusively for statistical and research purposes, i.e. scientific publications.

Section 1. General information

1. Year of birth _____________

2. Gender

□ Female □ Male

3. Where do you live? _________________ (please insert the town/city)

4. Education level (years): ________________

5. Do you live alone?

□ Yes □ No

5.a. If not, who lives with you?

□ Spouse
□ Daughter
□ Son
□ Grandchild
□ Other: ____________________

5.b. If you live alone, do you have a family member who lives in the same building or within walking distance?

□ Yes □ No

5.c. Who do you consider to be the person who provides the main informal source of care and support for you in relation to your diagnosis?

□ Spouse
□ Daughter
□ Son
□ Grandchild
□ Other: ____________________

6. Do you have any outside space in your home?

□ Balcony
□ Garden
□ Patio
□ Communal garden or yard shared with neighbours
□ Other: ____________________

7. Are you currently a smoker?

□ Yes □ No

7.a. If yes, how many cigarettes per day? __________________

8. Which type of devices do you have in your home? (Tick all that apply)

□ Smartphone
□ Mobile Phone /Landline phone
□ PC/computer
□ Television
□ Amazon devices (i.e. Alexa)
□ Tablet

Section 2. Individual protection methods

Please answer the following questions in relation to when the coronavirus outbreak started:

9. Have you bought a surgical mask or has someone given you one?

□ Yes □ No

9.a If not, why?

□ They sold out in my town/city
□ I don’t think I need one
□ They are too expensive
□ Other: _________________________

10. Have you bought (or has someone bought for you) disposable gloves?

□ Yes □ No

10.a If not, why?

□ They sold out in my town/city
□ I prefer to wash my hands
□ I use a disinfectant gel
□ Other: _______________

11. Do you know how long you should wash hands for them to be properly clean according to medical guidelines?

____ seconds Other: ____

11.b. Have you been washing your hands more often than before?

□ Yes □ No □ Maybe

Section 3. Knowledge about COVID-19

		Never	Rarely	Sometimes	Usually	Always
12	I’m trying to keep up to date with the latest information on coronavirus by watching TV, talk shows, reading magazines or searching for information on the internet					
13	I’m aware of the decisions and rules made by the Government					
14	I’m aware of the risks of coronavirus					
15	I’m aware of how the outbreak is spreading in different countries					
16	I’m trying to maintain social distancing and staying inside					

NOTE TO THE RESEARCHER: make sure you repeat the options to the patient for every question.

Section 4. How COVID-19 is affecting your life

17. Do you think that you contracted the coronavirus?

□ Yes □ No

17.a. If yes, did you contact your GP?

□ Yes □ No

17.b. Were you tested for the virus?

□ Yes □ No

17.c. Which symptoms did you have? ____

____

17.d. Other details: ____

____

18. Has anyone you know contracted the coronavirus?

□ Yes □ No

If yes, who: ____

19. Has anyone you know died because of contracting the coronavirus?

□ Yes □ No

If yes, who: ____

20. Due to the coronavirus, are you afraid to go out?

□ Yes, more than before
□ Slightly more than before
□ No more than usual
□ I was always afraid of going out

21. How many times a week do you:

□ Receive visits in your home from family or friends or caregivers: _________________
□ Go to the shops:___________________
□ Leave your property for a walk/run/bike ride: ________________
□ Drive your car: ______________
□ Go to the doctor/hospital/have a blood test: ______________
□ Go to the pharmacy: _________________
□ Buy a paper from the newsagent: _________________
□ Buy cigarettes from the shop: _______________
□ I never go out

22. How often, on average, do you go outside each day (garden, balcony, patio, out of the house etc.)?

□ Less than 3 hours □ More than 3 hours □ Other: __________________________

23. Have any of your routine medical visits been cancelled because of the coronavirus lockdown?

□ Yes □ No, I didn't have any booked □ No, they were not cancelled

If yes, please indicate which ones: ____

23.a. If yes, are you worried about it?

□ Yes □ No

If yes, why? ____

23.b. Are you taking any medications prescribed by a doctor?

□ Yes □ No

23.c. Have you had difficulties finding prescription medicines?

□ Yes □ No

23.d. If yes, please tick all that apply:

□ Difficulty obtaining a prescription from the doctor
□ The drugs are not available in the pharmacy
□ I need to wait for the pharmacy to order the drugs
□ I am avoiding going into the pharmacy because of the risk of coronavirus
□ Other: _______________________________

23.e. Have you forgotten to take your medication during the lockdown period?

□ Never □ Rarely □ Sometimes □ Often □ Always

23.f. Has this worsened since the lockdown began?

□ Yes □ No □ Maybe

23.g. Have you been able to contact your GP during the lockdown?

□ Yes, by phone
□ Yes, through video call
□ Yes, by text message
□ Yes, I went to the surgery
□ No, I have no medical concern
□ No, I don't want to bother them

23.h. Do you need specialist care for an ongoing chronic condition?

□ Yes □ No

If yes, please specify: ____

23.i. Have you been for any medical check-ups at the hospital/clinic/GP surgery since the start of the pandemic?

□ Yes □ No

If yes, please specify: ____

23.j. Are you concerned about any aspects of your health?

□ Yes □ No

If yes, please specify: ____

24. Did you have a formal or informal caregiver or family member who helped you perform any activities before the lockdown?

□ Yes □ I need one but didn't have one □ No, I do not need one, I am independent

24.a If yes, list the type of care that you received before the pandemic and during the lockdown:

	BEFORE PANDEMIC	DURING PANDEMIC/LOCKDOWN
Going to the toilet	YES/NO	YES/NO
Eating	YES/NO	YES/NO
Dressing	YES/NO	YES/NO
Personal hygiene	YES/NO	YES/NO
Walking	YES/NO	YES/NO
Bathing	YES/NO	YES/NO
Making phone calls	YES/NO	YES/NO
Grocery shopping	YES/NO	YES/NO
Cooking meals	YES/NO	YES/NO
Cleaning house	YES/NO	YES/NO
Doing the laundry	YES/NO	YES/NO
Driving	YES/NO	YES/NO
Taking medications	YES/NO	YES/NO
Help with financial tasks	YES/NO	YES/NO

25. Have your caregivers come to your home to see you during the lockdown?

□ They never come □ Less often
□ The same as before □ Not applicable, I live with them
□ More often

26. How do you keep in contact with friends and family members during the lockdown?

□ Phone calls □ Video calls □ Instant messages
□ They have visited me □ I have visited them

26.a. Has there been any change in your frequency of communication with family and friends since the coronavirus pandemic started?

□ More than usual □ Less than usual □ Unchanged

27. Have your shopping habits changed since the coronavirus pandemic started?

□ I buy only what I really need
□ I buy anything I feel like, whether I need it or not
□ I buy two or more packs of the same product
□ I buy less than before

28. Has your attitude to food changed since the coronavirus pandemic started?

□ I eat more healthier than before
□ I eat more unhealthy foods now
□ I snack a lot during the day
□ I eat less than before

29. If you're a smoker, has your smoking increased during the lockdown?

□ Yes □ No

29.a. If yes, how many more cigarettes/day compared to before: _________

30. Do you think that your weight has changed since the pandemic began?

□ Weight is the same □ Lost weight □ Gained weight

31. What kind of physical activity have you been doing during the pandemic?

□ Walking outside □ Cycling outside
□ Walking at home □ Exercises at home
□ None □ Other ________________________

31.a. How often have you been doing physical exercise during the pandemic?

_____ minutes per day
_____ times per week

Section 5. The effect of COVID-19 on emotional state

Please indicate the response the best describes how you feel at the moment, since the pandemic started

		Never	Rarely	Sometimes	Usually	Always
1	I'm unhappy doing so many things alone					
2	I lack companionship					
3	I find myself waiting for people to call or write more than before					
4	Some days I spend most of the day in bed					
5	I would love to hug someone					
6	The days seem to last too long					
7	People are around me but not with me					
8	I think that if something happens to me I won't have anyone who can help me					
9	I wish my family members would do more for me					
10	I have the feeling that there is someone in my house					
11	I feel angry					
12	I feel less motivated to do things					
13	I feel anxious					
14	I feel sad					
15	I worry that other people are not following social distancing measures					
16	I am worried about catching the coronavirus					
17	I am worried about getting into trouble for not following social distancing rules					
18	I am worried about my economic situation					
19	I have problems sleeping					
20	I treat myself with food or alcohol					
21	When the pandemic ends, I am afraid that I won't be able to resume my life as it was					
22	I'm trying to see the positive side of things, but it's difficult					
23	I don't think I can hold out much longer in this situation					

Thank you!

Appendix 2. The Effect of the COVID-19 Lockdown on Persons with Neurocognitive Disorders — Informant and Caregiver version

Identification code: ___________ **Date: ___ / ___ / ______**

THE EFFECT OF COVID-19 LOCKDOWN ON PERSONS WITH NEUROCOGNITIVE DISORDERS INFORMANT AND CAREGIVER VERSION

The purpose of this survey is to obtain information about your experience of the lockdown that has occurred because of the coronavirus pandemic. The questions are not designed to check up on you to see if you are following any rules - none of the information you provide will be given to anyone else. The questionnaire is completely anonymous and subject to the European and National privacy regulations (UE 2016/679). Your data will be used exclusively for statistical and research purposes, i.e. scientific publications.

NOTE TO THE RESEARCHER: This questionnaire should be administered to the person whom the patient identifies as being the main informal source of care and support in relation to their diagnosis (this can be a spouse, child, relative, friend or privately-hired support person who either lives or does not live with the patient. Please use the patient's name throughout the survey, in place of "*the patient*".

Section 1. General information

1. Year of birth _____________

2. Gender

□ Female □ Male

3. Where do you live? _________________ (please insert the town/city)

4. Education level (years): ________________

5. What relation are you to *the patient*?

□ Spouse

□ Daughter

□ Son

□ Grandchild

□ Other: ____________________

5.b. Do you consider yourself to be the person who provides the main informal source of care and support to *the patient* in relation to their diagnosis?

□ Yes □ No

6. Do you live with *the patient*?

□ Yes □ No

6.a. If not, do you live in the same building or nearby?

□ Yes □ No

6.b. Are you able to see him/her regularly?

□ Yes □ No

7. Are you currently a smoker?

□ Yes □ No

7.a. If yes, how many cigarettes per day? _________________

8. What devices do you have in your home? (Tick all that apply)

□ Smartphone
□ Mobile Phone /Landline phone
□ PC/computer
□ Television
□ Amazon devices (i.e. Alexa)
□ Tablet

8.a. Which type are in *the patient's* home? (Tick all that apply)

□ Smartphone
□ Mobile Phone /Landline phone
□ PC/computer
□ Television
□ Amazon devices (i.e. Alexa)
□ Tablet

8.b. During the lockdown, what type of devices have you used to communicate with *the patient*?

□ Phone call
□ Video call
□ Messages
□ Other: ____

Section 2. Individual protection methods

Please answer the following questions in relation to when the coronavirus outbreak started

9. Have you bought a surgical mask for yourself and/or *the patient*?

□ Yes □ No

9.a If not, why?

□ They sold out in my town/city
□ I don't think I need one
□ They are too expensive
□ Other: _________________________

10. Have you bought disposable gloves for yourself and/or *the patient*?

□ Yes □ No

10.a If not, why?

□ They sold out in my town/city
□ I prefer to wash my hands
□ I use disinfectant gel
□ Other: _______________

11. Are you making sure that *the patient* washes their hands more than before?

□ Yes □ No □ Maybe

11.a. Is it difficult to make him/her wash their hands?

□ No
□ He/she often forgets
□ He/she washes them several times because they forget that they have already washed them
□ Other: ____

Section 3. Knowledge about COVID-19

		Never	Rarely	Sometimes	Usually	Always
12	I'm trying to keep up to date with information about coronavirus by watching TV, talk shows, reading magazines or searching for information on the internet					
13	I'm aware of the decisions and rules made by the Government					
14	I'm aware of the risks of coronavirus					
15	I'm aware of how the outbreak is spreading in different countries					
16	I make sure that the patient is aware of the situation					
17	I'm following the Government's rules and recommendations about social distancing and staying inside					
18	I make sure that the patient follows the rules and recommendations					

NOTE TO THE RESEARCHER: make sure you repeat the options for every question

Section 4. How coronavirus is affecting your life

19. Do you think that you have contracted the coronavirus? □ Yes □ No

19.a. If yes, did you contact your GP? □ Yes □ No

19.b. Were you tested for the virus? □ Yes □ No

19.c. Which symptoms did you have? ____

____

19.d. Other details: ____

____

20. Has anyone you know contracted the coronavirus?

□ Yes □ No

If yes, who: ______________________________

21. Has anyone you know died because of contracting the coronavirus?

□ Yes □ No

If yes, who: ______________________________

22. Due to the coronavirus pandemic, have you lost your job?

□ Yes □ No

22.a. If not, has your company initiated any special working conditions?

□ Home-based working □ Other: ____________________

23. Due to the coronavirus, are you afraid to go out?

□ Yes, more than before □ No more than usual
□ Slightly more than before □ I was already afraid of going out

23.a. Are you afraid to visit *the patient* because of the coronavirus?

□ Yes □ No □ Not applicable

24. Have any of *the patient's* routine medical visits been cancelled because of the coronavirus pandemic and lockdown?

□ Yes □ No

If yes, please indicate which ones: ____

24.a. If yes, are you worried about it?

□ Yes □ No

If yes, why? ____

25. Does *the patient* take drugs prescribed by a doctor?

□ Yes □ No

25.a. If yes, does *the patient* take a drug for dementia?

□ Yes □ No

25.b. Have you experienced difficulties finding prescription medicines?

□ Yes □ No

25.c. If yes, please tick all that apply:

□ Difficulty obtaining a medical prescription from the doctor
□ The drugs are not available in the pharmacy
□ We need to wait for the pharmacy to order the drugs
□ We avoid going into the pharmacy because of the risk of coronavirus
□ Other: _________________________________

25.d. During the pandemic, how have you been monitoring *the patient's* medication intake?

□ Phone calls □ Messages
□ I give them directly □ Not applicable, they manage their drugs independently

25.e. Since the pandemic, does *the patient* ever forget to take their medicine?

□ Never □ Rarely □ Sometimes □ Usually □ Always

25.f. Has this gotten worse since before the pandemic started?

□ Yes □ No □ Maybe

25.g. Have you been able to contact your GP since the pandemic began?

□ Yes, by phone
□ Yes, through video call
□ Yes, by text message
□ Yes, I went to the surgery
□ No, I have no medical concern
□ No, I don't want to bother them

25.h. Do you or *the patient* need specialist medical care for an ongoing chronic condition?

□ Yes □ No

If yes, specify: ____

25.i. Since the lockdown began, have you attended medical check-ups at the hospital/clinic/GP surgery?

□ Yes □ No

If yes, please specify: ____

25.j. Are you concerned about any aspects of *the patient's* health?

□ Yes □ No

If yes, please specify: ____

26. Before the pandemic did you help *the patient* with any of their activities of daily living?

□ Yes □ No □ Not applicable, they are completely independent

26.a If yes, list the type of care that they received (from you or another informal caregiver) before the pandemic and during the lockdown:

	BEFORE PANDEMIC	DURING PANDEMIC/LOCKDOWN
Going to the toilet	YES/NO	YES/NO
Eating	YES/NO	YES/NO
Dressing	YES/NO	YES/NO
Personal hygiene	YES/NO	YES/NO
Walking	YES/NO	YES/NO
Bathing	YES/NO	YES/NO
Making phone calls	YES/NO	YES/NO
Grocery shopping	YES/NO	YES/NO
Cooking meals	YES/NO	YES/NO
Cleaning the house	YES/NO	YES/NO
Doing the laundry	YES/NO	YES/NO
Driving	YES/NO	YES/NO
Taking medication	YES/NO	YES/NO
Help with financial tasks	YES/NO	YES/NO

27. During the coronavirus pandemic, have you visited *the patient?*

□ Never
□ Less often
□ The same as before
□ Not applicable, I live with him/her
□ More often

28. During the coronavirus pandemic, how do you keep in contact with *the patient*?

□ Phone call □ Video call □ Instant messages
□ I've visited him/her □ He/she has visited me

28.a. During the coronavirus pandemic, how frequently have you been communicating with family and friends?

□ More than usual □ Less than usual □ Unchanged

29. Have your shopping habits changed since the coronavirus pandemic started?

□ I buy only what I really need
□ I buy anything I want, whether I need it or not
□ I buy two or more packs of the same product
□ I buy less than before

30. Has your attitude to food changed since the coronavirus pandemic began?

□ I eat more healthier than before
□ I eat more unhealthy foods now
□ I snack a lot during the day
□ I eat less than before

31. If you're a smoker, have you been smoking more during lockdown than before the pandemic?

□ Yes □ No

31.a. If yes, how many more cigarettes/day compared to before? _________

32. Do you think your weight has changed since the lockdown started?

□ Weight is the same □ Lost weight □ Gained weight

33. What physical activity have you been doing during the pandemic?

□ Walking outside
□ Cycling outside
□ Exercises at home
□ Walking at home
□ None
□ Other ______________________

33.a. How often do you exercise during the lockdown?

____ minutes per day
____ times per week

Section 5. The effect of COVID-19 on emotional state

Please indicate the response the best describe how you feel at the moment, since the pandemic started:

		Never	Rarely	Sometimes	Usually	Always
1	**The patient** asks for more help than he/she needs					
2	I'm tired of worrying about whether **the patient** is safe and following social distancing and hand-washing guidelines					
3	If something happens to **the patient** I don't know how to help him/her					
4	I'm afraid that the patient will tell me that everything is fine, but he/she isn't telling the truth					
5	**The patient** has told me that I have been abandoning them					
6	**The patient** doesn't appreciate the effort I make to take care of him/her despite the pandemic					
7	When **the patient** doesn't answer the phone, I think something has happened to him/her					
8	In some ways, we have had a better relationship since the pandemic began					
9	I feel I could do a better job in caring for **the patient**					
10	When the pandemic ends, I am afraid that the patient will not be able to resume **his/her** life in the same way that it was before					
11	I feel angry					
12	I feel burdened					
13	I feel anxious					
14	I feel sad					
15	I worry that other people are not following hygiene and social distancing measures					
16	I am worried about catching the coronavirus					
17	I am worried about getting into trouble for not following hygiene and social distancing measures					
18	I feel worried about my economic situation					
19	I have problems sleeping					
20	I treat myself to food and alcohol					
21	When the pandemic ends, I am afraid that I will not be able to resume my life as it was before					
22	I'm trying to see the positive side of things, but it's difficult					
23	I don't think I can hold out much longer in this situation					

Thank you!

Appendix 3. The Effect of the COVID-19 Lockdown on Persons with Neurocognitive Disorders – Italian Patient version

Codice paziente: ___________ **Data:** ___ / ___ / ____

GLI EFFETTI DELLA QUARANTENA SULLE PERSONE CON DISTURBI NEUROCOGNITIVI
VERSIONE PAZIENTE

Lo scopo di questo sondaggio è ottenere informazioni sulla Sua esperienza personale durante l'isolamento a causa della pandemia di coronavirus. Con questo sondaggio non vogliamo verificare se sta rispettando le regole, nessuna di queste informazioni verrà diffusa. Il questionario è completamente anonimo e soggetto alle normative sulla privacy europee e nazionali (UE 2016/679). I Suoi dati verranno utilizzati esclusivamente per finalità ricerca ed analisi statistiche, ad esempio pubblicazioni scientifiche.

Sezione 1. Informazioni generali

1. Anno di nascita _____________

2. Genere

□ Donna □ Uomo

3. Dove vive? _________________ (Inserire la città/paese)

4. Scolarità (in anni): ________

5. Vive da solo? □ Sì □ No

5.a. Se no, con chi vive?

□ Coniuge □ Nipote

□ Figlio □ Figlia

□ Altro: ____________________

5.b. Se vive da solo, ha un membro della Sua famiglia che vive con lei nello stesso edificio o a poca distanza?

□ Sì □ No

5.c. Chi è secondo lei la persona che le fornisce le maggiori cure e supporto di cui ha bisogno in relazione alla sua diagnosi?

□ Coniuge □ Nipote

□ Figlio □ Figlia

□ Altro: ____________________

6. Ha uno spazio esterno alla sua casa?

□ Balcone □ Giardino comunale o condominiale

□ Giardino □ Altro: ____________________

□ Terrazza

7. E' attualmente un fumatore?

□ Sì □ No

7.a. Se sì, quante sigarette al giorno: _________

8. Quali dispositivi ha a casa sua? (Indicare tutte le risposte valide)

□ Smartphone
□ Telefono cellulare/telefono fisso
□ PC/computer
□ Televisione
□ Amazon devices (i.e. Alexa)
□ Tablet

Sezione 2. Dispositivi di protezione individuale

Per favore risponda alle seguenti domande in riferimento al periodo di inizio dell'epidemia di coronavirus:

9. Ha comprato, o qualcuno lo ha fatto per lei, una mascherina?

□ Sì □ No

9.a. Se no, perché?

□ Sono introvabili nella mia città
□ Non penso di averne bisogno
□ Sono molto costose
□ Altro: ______________________

10. Ha comprato, o qualcuno lo ha fatto per lei, dei guanti usa e getta?

□ Sì □ No

10.a. Se no, perché?

□ Sono introvabili nella mia città
□ Preferisco lavarmi le mani più spesso
□ Ho un gel disinfettante
□ Altro: ______________________

11. Sa per quanto tempo ci si dovrebbe lavare le mani per farlo correttamente in accordo con le linee guida mediche?

_____ secondi Altro ______________________________

11.b. Si lava le mani molto più spesso di prima?

□ Sì □ No □ Forse

Sezione 3. Conoscenza del Covid-19

		Mai	Raramente	Qualche volta	Spesso	Sempre
12	Cerco di tenermi aggiornato seguendo telegiornali, seguendo talk show, leggendo riviste, acquistando libri sull'argomento					
13	Sono a conoscenza delle normative emanate dal mio Governo					
14	Conosco i rischi di contrarre il coronavirus					
15	Sono a conoscenza della situazione epidemiologica negli altri Paesi del mondo					
16	Cerco di mantenere la distanza sociale e rimanere a casa					

NOTA PER IL RICERCATORE: assicurarsi di ripetere le opzioni di risposta per ogni domanda.

Sezione 4. L'impatto del COVID-19 sulla vita quotidiana

17. Pensa di aver contratto il coronavirus?

□ Sì □ No

17.a. Se sì, ha contattato il medico di base?

□ Sì □ No

17.b. Si è sottoposto al tampone?

□ Sì □ No

17.c. Quali sintomi ha avuto? ____

____

17.d. Altri dettagli: ____

____

18. Qualcuno che conosce ha contratto il coronavirus?

□ Sì □ No

Se sì, chi? ____

19. Qualcuno che conosce è morto di coronavirus?

□ Sì □ No

Se sì, chi? ____

20. A causa del coronavirus, ha paura di uscire?

□ Sì, molto più di prima
□ Leggermente più di prima
□ Non più del solito
□ Ho sempre paura di uscire

21. Quante volte alla settimana:

□ Ricevo visite dei familiari o degli amici o del badante: ____________________
□ Fare compere: ____________________
□ Uscire per una passeggiata/corsa/bicicletta: ____________________
□ Guidare la propria auto: ____________________
□ Andare dal dottore/ospedale/centro analisi: ____________________
□ Andare in farmacia: ____________________
□ Comprare un giornale: ____________________
□ Acquistare le sigarette: ____________________
□ Non sono mai uscito di casa

22. Quanto spesso, in media, è uscito di casa ogni giorno (giardino, balcone, terrazzo o fuori casa)?

□ Meno di 3 ore □ 3/+ ore □ Altro ___________________

23. A causa delle misure restrittive, sono state cancellate alcune delle sue visite mediche di routine?

□ Sì □ No, non sono state cancellate □ Non ne avevo in programma

Se sì, per favore indicare quali: ____

23.a. Se sì, è preoccupato per questo?

□ Sì □ No

Se sì, perché? ____

23.b. Sta assumendo una terapia prescritta da un dottore?

□ Sì □ No

23.c. Ha avuto difficoltà nel reperimento dei farmaci prescritti?

□ Sì □ No

23.d. Se sì, inserire tutte le alternative possibili:

□ Difficoltà ad ottenere ricette mediche/rinnovo piano terapeutico
□ Le medicine non erano disponibili in farmacia
□ Ho dovuto aspettare l'ordine in farmacia
□ Ho evitato di andare in farmacia per evitare le occasioni di contagio
□ Altro: ___________________________________

23.e. In questo periodo, si è dimenticato di assumere le sue medicine?

□ Mai □ Raramente □ Qualche volta □ Spesso □ Sempre

23.f. Questo aspetto è peggiorato rispetto a prima dell'inizio dell'isolamento?

□ Sì □ No □ Forse

23.g. Durante l'isolamento, è riuscito a contattare il suo medico di base?

□ Sì, telefonicamente □ Sì, sono andata presso lo studio
□ Sì, videochiamata □ No, non ho avuto problemi di salute
□ Sì, messaggi □ No, ho preferito non disturbarlo

23.h. Ha avuto bisogno di cure specialistiche per una condizione cronica in corso?

□ Sì □ No

Se sì, specificare: ____

23.i. Durante l'isolamento, ha partecipato a controlli medici presso l'ospedale / clinica / studio medico:

□ Sì □ No

Se sì, specificare: ____

23.j. E' preoccupato per qualche aspetto della sua salute?

□ Sì □ No

Se sì, quale e perché ____

24. Prima dell'isolamento, aveva un familiare o un assistente formale/informale che l'aiutava nello svolgimento delle sue attività?

□ Sì □ No □ N/A autonomo

24. a. Se sì, indicare quale tipo di aiuto ha ricevuto prima e durante il periodo di isolamento:

	PRIMA LOCKDOWN	DURANTE LOCKDOWN
Andare in bagno	SI/NO	SI/NO
Alimentazione	SI/NO	SI/NO
Vestirsi	SI/NO	SI/NO
Cura personale	SI/NO	SI/NO
Camminare	SI/NO	SI/NO
Fare la doccia/bagno	SI/NO	SI/NO
Telefonare	SI/NO	SI/NO
Fare acquisti	SI/NO	SI/NO
Cucinare	SI/NO	SI/NO
Pulizie di casa	SI/NO	SI/NO
Fare il bucato	SI/NO	SI/NO
Guidare	SI/NO	SI/NO
Assumere farmaci	SI/NO	SI/NO
Finanza	SI/NO	SI/NO

25. Dall'inizio dell'epidemia di coronavirus, il suo caregiver è venuto a trovarla?

□ Non sono mai venuti □ Meno spesso
□ Come al solito □ Più spesso
□ N/A Vivo con loro

26. Dall'inizio dell'epidemia di coronavirus, come si è tenuto in contatto con parenti e amici?

□ Telefonate □ Video chiamate □ Messaggi
□ Sono andato a trovarli □ Sono venuti a trovarmi

26.a. Dall'inizio dell'epidemia di coronavirus, ci sono stati dei cambiamenti nella frequenza delle comunicazioni con parenti e amici?

□ Aumentata □ Ridotta □ Non è cambiata

27. Dall'inizio dell'epidemia di coronavirus, ha cambiato il suo modo di fare la spesa?

□ Cerco di comprare soltanto ciò che mi serve realmente
□ Acquisto qualsiasi cosa, senza chiedermi se sia necessaria
□ Ho comprato due o più confezioni dello stesso prodotto
□ Ho fatto meno acquisti

28. Dall'inizio dell'epidemia di coronavirus, sono cambiate le sue abitudini alimentari?

□ Mangio molto più salutare di prima
□ Mangio meno sano di prima
□ Faccio numerosi spuntini durante il giorno
□ Mangio meno di prima

29. Se fumatore, durante il periodo di isolamento ha fumato di più?

□ Sì □ No

29.a. Se sì, quante sigarette/giorno rispetto a prima: ________

30. Pensa che il suo peso sia cambiato dall'inizio dell'epidemia?

□ Il peso è lo stesso □ Ho perso peso □ Il peso è aumentato

31. Durante l'isolamento, quale tipo di attività fisica ha condotto?

□ Passeggiare
□ Esercizi a casa
□ Nessuno
□ Andare in bicicletta
□ Camminare in casa
□ Altro _______________________

31.a. Durante l'epidemia di coronavirus, con quale frequenza ha condotto gli esercizi?

_____ minuti al giorno
_____ volte a settimana

Sezione 5. Gli effetti del COVID-19 sullo stato emotivo

Per favore indicare la risposta che meglio descrive come si sente in questo periodo, da quando è iniziata l'epidemia

		Mai	Raramente	Qualche volta	Spesso	Sempre
1	Mi dispiace fare molte cose da solo					
2	Mi manca la compagnia					
3	Mi ritrovo ad aspettare che qualcuno mi chiami o mi mandi un messaggio molto più di prima					
4	Certi giorni sono così noiosi da trascorrerli a letto					
5	Avrei voglia di abbracciare qualcuno					
6	Le giornate sembrano non finire mai					
7	Le persone sono intorno a me, ma non con me					
8	Credo che se dovesse succedermi qualcosa non avrei nessuno a cui poter chiedere aiuto					
9	Vorrei che i miei familiari facessero molto di più per me					
10	Ho la sensazione che ci sia qualcuno a casa con me					
11	Sono irritabile					
12	Mi sento meno motivato					
13	Mi sento ansioso					
14	Mi sento triste					
15	Sono preoccupato che gli altri non rispettino le regole anti contagio e di isolamento					
16	Sono preoccupato di contrarre il coronavirus					
17	Sono preoccupato di subire provvedimenti se non rispetto le regole anti-contagio					
18	Sono preoccupato della mia situazione economica					
19	Ho difficoltà a dormire					
20	Mi concedo qualche extra in più (cibo, bevande alcoliche)					
21	Quando tutto finirà, temo di non riuscire a ritornare alla mia vita precedente con facilità					
22	Sto cercando di vedere il lato positivo, ma non è per niente semplice					
23	Non credo che potrò sopportare tutto questo per molto tempo					

Grazie!

Appendix 4. The Effect of the COVID-19 Lockdown on Persons with Neurocognitive Disorders – Italian Caregiver version

Codice paziente: ___________ **Data: ___ / ___ / ___**

GLI EFFETTI DELLA QUARANTENA SULLE PERSONE CON DISTURBI NEUROCOGNITIVI VERSIONE CAREGIVER

Lo scopo di questo sondaggio è ottenere informazioni sulla Sua esperienza personale durante l'isolamento a causa della pandemia di coronavirus. Con questo sondaggio non vogliamo verificare se sta rispettando le regole, nessuna di queste informazioni verrà diffusa. Il questionario è completamente anonimo e soggetto alle normative sulla privacy europee e nazionali (UE 2016/679). I Suoi dati verranno utilizzati esclusivamente per finalità ricerca ed analisi statistiche, ad esempio pubblicazioni scientifiche.

NOTA PER IL RICERCATORE: Questo questionario deve essere somministrato alla persona che il paziente identifica come la principale fonte informale di assistenza e supporto in relazione alla sua diagnosi (può essere un coniuge, un figlio, un parente, un amico o un sostegno assunto da privati che vive o non vive con il paziente). Si prega di utilizzare il nome del paziente durante il questionario, al posto di "IL PAZIENTE".

Sezione 1. Informazioni generali

1. Anno di nascita _____________

2. Genere

□ Donna □ Uomo

3. Dove vive? _________________ (Inserire la città/paese)

4. Scolarità (in anni): ________

5. Qual è il suo grado di relazione col *paziente*?

□ Figlio □ Coniuge
□ Figlia □ Nipote
□ Altro: _________________________

5.b. Ritiene di essere la persona che si occupa principalmente di fornire le cure informali e il supporto al *paziente* in relazione alla sua diagnosi?

□ Sì □ No

6. Vive con il *paziente*?

□ Sì □ No

6.a. Se no, vive nello stesso palazzo o a poca distanza?

□ Sì □ No

6.b. Ha la possibilità di vederlo regolarmente?

□ Sì □ No

7. E' attualmente un fumatore? □ Sì □ No

7.a. Se sì, quante sigarette/giorno: _________

8. Quali dispositivi ha a casa sua? Indicare tutte le risposte valide.

□ Smartphone
□ Telefono cellulare/telefono fisso
□ PC/computer
□ Televisione
□ Amazon devices (i.e. Alexa)
□ Tablet

8.a. Quali dispositivi sono a casa del *paziente*? Indicare tutte le risposte valide.

□ Smartphone
□ Telefono cellulare/telefono fisso
□ PC/computer
□ Televisione
□ Amazon devices (i.e. Alexa)
□ Tablet

8.b. Durante l'isolamento, quale modalità di comunicazione ha usato con il *paziente*? Indicare tutte le risposte valide.

□ Telefonata
□ Videochiamata
□ Messaggi
□ Altro: ____

Sezione 2. Dispositivi di protezione individuale

Per favore risponda alle seguenti domande in riferimento al periodo di inizio dell'epidemia di coronavirus:

9. Ha comprato una mascherina per sé e/o per il *paziente*?

□ Sì □ No

9.a. Se no, perché?
□ Sono introvabili nella mia città
□ Non penso di averne bisogno
□ Sono molto costose
□ Altro: ____

10. Ha comprato dei guanti usa e getta per sé e/o per il *paziente*?

□ Sì □ No

10.a. Se no, perché?

□ Sono introvabili nella mia città
□ Preferisco lavarmi le mani
□ Ho un gel disinfettante
□ Altro: ____

11. Si assicura molto più di prima che il *paziente* si sia lavato le mani?

□ Sì □ No □ Forse

11.a. E' difficile far lavare le mani al *paziente*?

□ No
□ Si dimentica spesso di farlo
□ Si lava diverse volte le mani perché si dimentica di averlo già fatto
□ Altro: ____

Sezione 3. Conoscenza del COVID-19

		Mai	Raramente	Qualche volta	Spesso	Sempre
12	Cerco di tenermi aggiornato seguendo telegiornali, seguendo talk show, leggendo riviste, acquistando libri sull'argomento					
13	Sono a conoscenza delle normative emanate dal Governo					
14	Conosco i rischi di contrarre il coronavirus					
15	Sono a conoscenza della situazione epidemiologica negli altri Paesi					
16	Mi assicuro che il paziente sia al corrente della situazione					
17	Cerco di rispettare le normative del Governo o le indicazioni sul distanziamento sociale e di rimanere a casa					
18	Mi assicuro che il paziente rispetti le misure di contenimento					

NOTA PER IL RICERCATORE: assicurarsi di ripetere le opzioni di risposta per ogni domanda.

Sezione 4. L'impatto del coronavirus sulla vita quotidiana

19. Pensa di aver contratto il coronavirus? □ Sì □ No

19.a. Se sì, ha contattato il suo medico di base? □ Sì □ No

19.b. Si è sottoposto al tampone? □ Sì □ No

19.c. Quali sintomi ha avuto? ____

____

19.d. Altri dettagli: ____

____

20. Qualcuno che conosce ha contratto il coronavirus?

□ Sì □ No

Se sì, chi? ____

21. Qualcuno che conosce è morto di coronavirus?

□ Sì □ No Se sì, chi? ____

22. A causa dell'epidemia di coronavirus, ha perso il suo lavoro?

□ Sì □ No

22.a. Se no, la sua azienda ha avviato delle procedure di lavoro speciali?

□ Lavoro agevole □ Cassa integrazione □ Altro: ________________________

23. A causa del coronavirus, ha paura di uscire?

□ Sì, molto più di prima
□ Non più del solito
□ Leggermente più di prima
□ Ho sempre paura di uscire

23.a. A causa del coronavirus, ha paura di andare a trovare il *paziente*?

□ Sì □ No □ Non applicabile

24. A causa della pandemia di coronavirus e delle misure restrittive, sono state cancellate alcune visite mediche di routine del *paziente*?

□ Sì □ No

Se sì, indicare quali: ____

24.a E' preoccupato per questo?

□ Sì □ No

Se sì, perché? ____

25. Il *paziente* assume una terapia farmacologica prescritta da un medico?

□ Sì □ No

25.a. Se sì, assume un farmaco per la demenza?

□ Sì □ No

25.b. È stato difficile reperire i farmaci?

□ Sì □ No

25.c. Se sì, inserire tutte le alternative possibili:

□ Difficoltà ad ottenere ricette mediche/rinnovo piano terapeutico
□ Le medicine non erano disponibili in farmacia
□ Ho dovuto aspettare l'ordine in farmacia
□ Ho evitato di andare in farmacia per evitare le occasioni di contagio
□ Altro: ____

25.d. Durante l'epidemia, come ha monitorato l'assunzione dei farmaci da parte del *paziente*?

□ Telefonata □ Non ha bisogno di aiuto

□ Messaggio □ Glieli do io

25.e. Dall'inizio dell'epidemia, il *paziente* si è dimenticato di assumere le medicine?

□ Mai □ Raramente □ Qualche volta □ Spesso □ Sempre

25.f. Questo aspetto è peggiorato rispetto a prima della pandemia?

□ Sì □ No □ Forse

25.g. Durante l'epidemia, le è stato possibile contattare il medico di base?
□ Sì, telefonicamente
□ Sì, videochiamata
□ Sì, messaggi
□ Sì, sono andata presso lo studio
□ No, non ho avuto problemi di salute
□ No, ho preferito non disturbarli

25.h. Ha avuto bisogno di cure specialistiche per sé o per il *paziente* per una condizione cronica?

□ Sì □ No

Se sì, specificare: ____

25.i. Dall'inizio dell'isolamento, ha effettuato controlli medici presso l'ospedale / clinica / studio medico?

□ Sì □ No

Se sì, specificare: ____

25.j. E' preoccupato per qualche aspetto della salute del *paziente*?

□ Sì □ No

Se sì, quale e perché ____

26. Prima dell'epidemia, aiutava l'anziano nello svolgimento delle sue attività basiche e strumentali della vita quotidiana?

□ Sì □ No □ N/A autonomo

26.a. Se sì, indicare su quali attività il paziente ha ricevuto supporto da parte sua o di un altro caregiver prima e dopo l'isolamento:

	PRIMA LOCKDOWN	DURANTE LOCKDOWN
Andare in bagno	SI/NO	SI/NO
Alimentazione	SI/NO	SI/NO
Vestirsi	SI/NO	SI/NO
Cura personale	SI/NO	SI/NO
Camminare	SI/NO	SI/NO
Fare la doccia/bagno	SI/NO	SI/NO
Telefono	SI/NO	SI/NO
Fare acquisti	SI/NO	SI/NO
Cucinare	SI/NO	SI/NO
Pulizie di casa	SI/NO	SI/NO
Fare il bucato	SI/NO	SI/NO
Guidare	SI/NO	SI/NO
Assumere farmaci	SI/NO	SI/NO
Finanza	SI/NO	SI/NO

27. Durante l'epidemia di coronavirus, è andato a trovare l'anziano?

□ Mai □ Meno spesso
□ Come al solito □ Più spesso
□ N/A Vivo con lui

28. Durante l'epidemia di coronavirus, come si è tenuto in contatto con il *paziente*?

□ Telefonate □ Video chiamate □ Messaggi
□ Sono andato a trovarlo □ E' venuto a trovarmi

28.a Durante l'epidemia di coronavirus, con quale frequenza ha comunicato con amici e parenti?

□ Aumentata □ Ridotta □ Non è cambiata

29. Dall’inizio dell’epidemia di coronavirus, ha cambiato il suo modo di fare la spesa?

□ Cerco di comprare soltanto ciò che mi serve realmente
□ Acquisto qualsiasi cosa senza chiedermi se sia necessaria
□ Ho comprato due o più confezioni dello stesso prodotto
□ Ho fatto meno acquisti di prima

30. Dall’inizio dell’epidemia di coronavirus, sono cambiate le sue abitudini alimentari?

□ Mangio molto più salutare di prima
□ Mangio meno sano di prima
□ Faccio numerosi spuntini durante il giorno
□ Mangio meno di prima

31. Se fumatore, durante il periodo di isolamento ha fumato di più rispetto a prima della pandemia?

□ Sì □ No

31.a Se sì, quante sigarette/giorno rispetto a prima: ________

32. Pensa che il suo peso sia cambiato dall’inizio dell’epidemia?

□ Il peso è lo stesso □ Ho perso peso □ Il peso è aumentato

33. Durante l’isolamento, quale tipo di attività fisica ha condotto?

□ Passeggiare
□ Esercizi a casa
□ Nessuno
□ Andare in bicicletta
□ Camminare in casa
□ Altro ______________________

33.a. Durante l’epidemia da coronavirus, con quale frequenza ha condotto gli esercizi?

____ minuti al giorno
____ volte a settimana

Sezione 5. Gli effetti del COVID-19 sullo stato emotivo

Per favore indicare la risposta che meglio descrive come si sente in questo periodo, da quando è iniziata l'epidemia

		Mai	Raramente	Qualche volta	Spesso	Sempre
1	Il **paziente** mi chiede molto più aiuto di prima					
2	Sono stanco di preoccuparmi che **il paziente** rispetti la quarantena					
3	Se dovesse succedergli qualcosa **(al paziente)**, non saprei come aiutarlo					
4	Sono preoccupato che **il paziente** mi dica che va tutto bene, ma in realtà mi nasconda qualcosa					
5	**Il paziente** mi accusa di averlo lasciato solo					
6	**Il paziente** non apprezza i miei sforzi nel prendermi cura di lui nonostante gli impedimenti della quarantena					
7	Quando **il paziente** non risponde subito al telefono, penso gli sia successo qualcosa					
8	Per certi versi, da quando sono iniziate le misure restrittive il nostro rapporto è migliorato					
9	Penso che potrei fare di meglio per **il paziente** di cui mi prendo cura					
10	Quando finirà tutto, sono preoccupato che **il paziente** non riuscirà a ricominciare la sua vita così com'era					
11	Sono irritabile					
12	Sono stressato					
13	Mi sento ansioso					
14	Mi sento triste					
15	Sono preoccupato che gli altri non rispettino le regole anti contagio e di isolamento					
16	Sono preoccupato di contrarre il coronavirus					
17	Sono preoccupato di subire provvedimenti se non rispetto le regole anti-contagio					
18	Sono preoccupato della mia situazione economica					
19	Ho difficoltà a dormire					
20	Mi concedo qualche extra in più (cibo, bevande alcoliche)					
21	Non credo che potrò sopportare tutto questo per molto tempo					
22	Sto cercando di vedere il lato positivo, ma non è per niente semplice					
23	Vorrei che tutto questo non fosse vero					

Grazie!
